# Effectiveness of influenza and pneumococcal vaccination in frail older adults

**DOI:** 10.1590/1806-9282.20250884

**Published:** 2026-03-30

**Authors:** Daniela Castelo Azevedo, Estevão Alves Valle, Débora Cerqueira Calderaro

**Affiliations:** 1 Universidade Federal de Minas Gerais, Faculty of Medicine – Belo Horizonte (MG), Brazil.; 2 Mais 60 Saúde – Belo Horizonte (MG), Brazil.

**Keywords:** Frail elderly, Pneumococcal vaccines, Influenza vaccines, Hospitalization

## Abstract

**OBJECTIVE:**

The aim of the study was to determine whether vaccinating older adults with PCV13 (pneumococcal conjugate vaccine) and/or PPSV23 (pneumococcal polysaccharide vaccine) and/or the influenza vaccine can reduce pneumonia-related hospitalizations and all-cause death.

**METHODS:**

Retrospective cohort of subjects ≥60 years who attended an outpatient clinic in Minas Gerais, Brazil (May 1, 2021 to April 26, 2024). Data regarding pneumonia hospitalizations, all-cause death, pneumococcal and influenza vaccination, demographic, and clinical characteristics were collected from an electronic health record (data in the cloud were available upon request). Only patients with valid data were included and were divided into groups based on their vaccination history, pneumococcal vaccinations (PVC13 and/or PPSV23), influenza vaccination, pneumococcal+influenza vaccinations, and unvaccinated, to calculate the association of vaccination with pneumonia hospitalizations and all-cause death by Cox proportional hazard models, adjusted for demographic and clinical factors.

**RESULTS:**

A total of 12,268 older adults were analyzed, with a median age of 79 (interquartile range 72–86), and 73% were female. Vaccination rates were 56% for influenza, 29% for pneumococcal vaccines, and 24% for both influenza and pneumococcal vaccines. Neither pneumococcal nor influenza vaccination alone showed a protective effect against pneumonia hospitalization. The combination of pneumococcal and influenza vaccines was associated with a 35% reduction in pneumonia hospitalization (HR 0.65; 95%CI 0.51–0.83). There was no association between vaccination and allcause death, although the influenza vaccine tended to reduce this risk (HR 0.68; 95%CI 0.46–1.00; p-value 0.053).

**CONCLUSION:**

Despite low vaccination rates, the combination of influenza and pneumococcal vaccines reduces pneumonia hospitalizations among older adults by 35%.

## INTRODUCTION

Pneumococcal disease poses a public health concern world-wide^1^. It is responsible for invasive and non-invasive clinical infections and is the leading cause of pneumonia^2^. In Brazil, lower respiratory tract infections, including community-acquired pneumonia (CAP), ranked as the third leading cause of mortality in both 1990 and 2015, particularly impacting young children and older adults^3^.

To prevent CAP, the Brazilian Ministry of Health recommends vaccinations against influenza and pneumococcus^4^. The National Health System (“Sistema Único de Saúde—SUS”) currently offers influenza vaccination to adults ≥60 annually. In relation to pneumococcal vaccination, the SUS provides the scheme with the pneumococcal conjugate vaccine (PCV13), followed by the pneumococcal polysaccharide vaccine (PPSV23), only to individuals at high risk for pneumococcal disease and invasive pneumococcal disease, such as those living with human immunodeficiency virus/acquired immunodeficiency syndrome (HIV/AIDS) or cancer^5^. However, vaccination can be done in private clinics if the patient can afford it. The Brazilian Society of Immunizations (SBIm), a non-profit scientific entity not affiliated with the SUS, brings together professionals from various specialties interested in vaccination and advocates for a routine pneumococcal vaccination scheme for adults over 60 years. This scheme consists of one dose of PCV20 or a sequential administration of PCV15 or PCV13 followed by PPSV23, 6–12 months later and again 5 years after the first PPSV23 dose^6^.

Given the risks associated with pneumococcal disease and the limited access to pneumococcal vaccination for most older adults in Brazil, this real-world retrospective cohort study aims to determine whether vaccinating this population with PCV13 and/or PPSV23, alongside the influenza vaccine, can reduce pneumonia hospitalizations and all-cause death.

## METHODS

### Study design and data source

This is a real-world data retrospective cohort study that utilized data from a digital platform (LifeCode, EFG Inteligencia e Consultoria Ltda, Belo Horizonte, Brazil). The trained health professionals have utilized this platform to document all clinic visits and telehealth services provided to community-dwelling older adults (≥60 years old) affiliated with a geriatric outpatient network of clinics in Belo Horizonte, Southeastern Brazil. The data spans from April 1, 2021 to April 26, 2024. The platform offers an electronic health record (EHR) with structured entry forms for clinical information (diagnosis, current medications, presenting symptoms, and vaccination history). This system facilitates patient follow-up by a multidisciplinary team (physicians, nurses, and physical therapists. All data is securely stored in a cloud environment and is available to researchers upon request.

We included all patients with valid data on hospitalizations, all-cause death, pneumococcal and influenza vaccination, demographic, and clinical characteristics during the study period. Patients without this available data were not included in the study database. The local Ethics Committee granted ethical approval, and a waiver for informed consent was obtained (CEP 5.579.944 and CAAE 58545422.5.0000.8787).

### Assessment of outcomes and covariates

All hospitalizations were reported by health insurance, and the nursing team contacted the patients or their families within 48 h of the event. Hospitalization details, such as causes, duration, and treatments, were uploaded to the EHR, along with hospital discharge documentation.

The health insurance systems also automatically reported death occurrences, which the assisting team registered in the EHR.

Immunization cards are requested for all patients at the clinic entrance and at every outpatient appointment with the healthcare team to guide patients on their up-to-date vaccinations. Vaccinations are recorded in a specific field in the EHR. The vaccination registration for Influenza, PCV13, and/or PPSV23 in the EHR was considered positive. PCV15 and PVC20 vaccination, approved for commercialization in Brazil in September 2023 and February 2024, respectively, were not considered in this study since there were still no records of patients vaccinated with these vaccines.

Age, multimorbidity, frailty, sex, chronic obstructive pulmonary disease (COPD), current smoking, and immunosuppression at the study entry were considered potential confounding factors for the association between influenza and/or pneumococcal vaccination and pneumonia hospitalization and all-cause death.

Multimorbidity, which refers to the co-occurrence of multiple diseases, is a reliable measure in primary care and frequently guides epidemiologic studies^7^. We assessed multimorbidity by counting the number of medical diagnoses. This method predominates in primary healthcare research^8,9^.

Frailty was assessed by the Clinical-Functional Vulnerability Index-20 (IVCF-20), a reliable and validated instrument^10^. It consists of 20 closed-ended questions divided into eight sections: age, health self-perception, functional disabilities, cognition, mood, mobility, communication, and multiple comorbidities. Each section has a specific score, with a maximum of 40 points. A higher score indicates a greater risk of clinical-functional vulnerability in older adults^10^. The IVCF-20 is administered upon patient admission to the clinic and annually during follow-up. In this study, we used the IVCF closest to the study baseline.

COPD was assessed by medical diagnosis. Current smoking recorded in EHR was registered. A patient was considered immunosuppressed if they had any current cancer, HIV/AIDS, a transplant, or other conditions described in your EHR according to the SUS definition for very high risk of pneumococcal disease (PD) and invasive pneumococcal disease (IPD)^5^.

### Statistical analysis

Descriptive analyses included frequencies and percentages (%) for categorical variables and medians and interquartile ranges (IQRs) for continuous variables since the distribution was not normal.

We used Cox proportional hazard models to investigate the association between pneumococcal (PCV13 and/or PPSV23) and/or influenza vaccination and the time to first pneumonia-associated hospitalization and all-cause death. Three possibilities of vaccination status were considered in the analysis: pneumococcal vaccination only (PCV13 and/or PPSV23), influenza vaccination only, and both vaccines, taking as reference the absence of vaccination. Pneumococcal and influenza vaccination status were time-varying covariates. Persons were deemed pneumococcal vaccinated 14 days after receiving the vaccine. For influenza vaccination, individuals were considered vaccinated 14 days post-administration and remained so through the end of the corresponding year. The multivariate models were adjusted to covariates at the study entry. Model 0 examined the association of vaccination status with pneumonia-related hospitalization and all-cause deaths without any adjustments, while model 1 was adjusted for age and sex, model 2 was adjusted for model 1 plus frailty and multimorbidity, and model 3 was adjusted for model 2 plus COPD, immunosuppression, and current smoking. The associations between vaccination status and pneumonia-associated hospitalization and all-cause death were measured using the hazard ratio (HR) and its 95%CI.

Analyses were performed in STATA (version 14.0, StataCorpLP, College Station, USA). Significance was set considering a 95% confidence level (α=5%).

## RESULTS

Data from 12,268 older adults were analyzed. They presented a median age of 79 (interquartile range [IQR] 72–86), and 8,907 (73%) were female. The pneumonia hospitalization incidence was 16.1 per 1,000 person-years (95%CI 14.7–17.6), and the all-cause death rate was 6.1 per 1,000 person-years (95%CI 5.3–7.0)—the median survival time until these events was not reached. The influenza vaccine was received by 6,912 (56%) individuals, and pneumococcal vaccines by 3,563 (29%), with PCV13 by 1,665 (13.6%) and PPSV23 by 1,898 (15.4%) individuals.

Table 1 demonstrates the characteristics of patients according to pneumococcal and/or influenza vaccination status. Patients vaccinated with both the pneumococcal and influenza vaccines were older and presented a more significant morbidity burden and a higher prevalence of COPD and immunosuppression.

**Table 1:** Socio-demographic characteristics, pneumonia-associated hospitalization, and all-cause death according to the vaccination status.

Characteristics	Total sample (n=12,268)	Unvaccinated (n=4,709)	Only influenza (n=3,996)	Pneumococcal+influenza vaccine (n=2,916)	Only pneumococcal (n=647)
Median age, years (IQR)	79 (72–86)	78 (70–85)	79 (71–86)	81 (74–87)	81 (74–88)
Female sex, n (%)	8,907 (73)	3,409 (72)	2,916 (73)	2,134 (73)	478 (74)
Multimorbidity, median (IQR)	5 (3–8)	4 (2–6)	5 (3–8)	7 (4–9)	6 (4–9)
Frailty score, median (IQR)	14 (10–21)	14 (10–21)	14 (9–20)	14 (10–21)	16 (11–23)
Current smoking, n (%)	387 (3.2)	145 (3)	131 (3.3)	94 (3.2)	17 (2.6)
COPD, n (%)	485 (4.0)	146 (3)	129 (3.2)	177 (6)	33 (5.1)
Immunosuppressed, n (%)	856 (7.0)	224 (4.8)	260 (6.5)	318 (11)	54 (6.3)
Pneumonia-associated hospitalization (%)	492 (4.0)	150 (3.2)	167 (4.2)	138 (4.7)	37 (5.7)
All cause-deaths 9%)	187 (1.52)	58 (1.23)	50 (1.2)	56 (1.9)	23 (3.5)

IQR: interquartile range; COPD: chronic obstructive pulmonary disease.

Table 2 describes the results of the crude model 0 and multivariable models (1–3) evaluating the association between vaccination status and pneumonia-associated hospitalization. After all adjustments (model 3), the isolated pneumococcal or influenza vaccination did not show a protective effect, while the combined pneumococcal and influenza vaccination was associated with a 35% risk reduction of pneumonia-associated hospitalization (HR 0.65; 95%CI 0.51–0.83).

**Table 2:** Risk of pneumonia-associated hospitalization according to pneumococcal and influenza vaccination status^*^.

Pneumococcal and influenza vaccination status	Pneumonia-associated hospitalization				
	Model 0 HR (95%CI)	Model 1 HR (95%CI)	Model 2 HR (95%CI)	Model 3 HR (95%CI)	p-value
Unvaccinated	REF	REF	REF	REF	
Pneumococcal vaccine	0.89 (0.62–1.27)	0.75 (0.52–1.07)	0.73 (0.51–1.05)	0.73 (0.51–1.05)	0.099
Influenza vaccine	0.69 (0.55–0.86)	0.74 (0.59–0.93)	0.82 (0.65–1.02)	0.82 (0.66–1.03)	0.094
Pneumococcal+influenza vaccine	0.59 (0.47–0.75)	0.60 (0.48–0.76)	0.65 (0.52–0.83)	0.65 (0.51–0.82)	0.000

*The risk of pneumonia-associated hospitalization was measured by the hazard ratio (HR) and its respective confidence intervals (CI) for vaccinated subjects with the pneumococcal vaccine (PVC13 and/or PPSV23), the Influenza vaccine, and the pneumococcal+influenza vaccine as compared with unvaccinated subjects. The model 0 was the crude risk, without adjustment. The multivariate HR was adjusted by age and sex (Model 1); by age, sex, multimorbidity, and frailty (Model 2); and by age, sex, multimorbidity, and frailty plus current smoking, COPD, and immunosuppression (Model 3). HR: hazard ratio; CI: confidence interval.

Table 3 shows the results of the model 0 and multivariable models (1–3) analyzing the association between vaccination status and all-cause death. There was no association between pneumonia and influenza vaccination status and all-cause deaths, although the influenza vaccine shows a tendency to reduce the risk of death—HR (95%CI): 0.68 (0.46–1.00); p-value 0.053.

**Table 3:** Risk of all-cause death according to pneumococcal and influenza vaccination status^*^.

Pneumococcal and influenza vaccination status	All-cause death				
	Model 0 HR (95%CI)	Model 1 HR (95%CI)	Model 2 HR (95%CI)	Model 3 HR (95%CI)	p-value
Unvaccinated	REF	REF	REF	REF	
Pneumococcal vaccine	1.51 (0.93–2.45)	1.28 (0.79–2.09)	1.21 (0.74–1.98)	1.21 (0.74–1.98)	0.438
Influenza vaccine	0.55 (0.37–0.80)	0.59 (0.40–0.87)	0.67 (0.45–0.98)	0.68 (0.46–1.00)	0.053
Pneumococcal+influenza vaccine	0.65 (0.45–0.95)	0.67 (0.46–0.97)	0.70 (0.48–1.03)	0.71 (0.49–1.04)	0.083

*The risk of all-cause death was measured by the hazard ratio (HR) and its respective confidence intervals (CI) for vaccinated subjects with the pneumococcal vaccine (PVC13 and/or PPSV23), the influenza vaccine, and the pneumococcal+Influenza vaccine as compared with unvaccinated subjects. The model 0 was the crude risk, without adjustment. The multivariate HR was adjusted by age and sex (Model 1); by age, sex, multimorbidity, and frailty (Model 2), and by age, sex, multimorbidity, and frailty plus current smoking, COPD, and immunosuppression (Model 3). HR: hazard ratio; CI: confidence interval.

## DISCUSSION

In this retrospective cohort study examining data from community frail older adults, it was found that pneumococcal plus influenza vaccinations reduced the risk of pneumonia hospitalization but did not decrease all-cause death. The influenza vaccine was noted to potentially reduce the risk of death. The study also pointed out concerns regarding vaccination coverage, as only 24% of participants had received both vaccines, 56% had received the influenza vaccine, and 29% had received the pneumococcal vaccine.

Observational studies support the finding that the pneumococcal plus influenza vaccinations reduce pneumonia-related hospitalizations and prevent all-cause death in older patients. An Italian cohort study involving older adults (mean age of 75.7) found influenza reduced all-cause death by 13%, with stronger effects when combined with pneumococcal vaccination^11^. A meta-analysis with adults ≥65 shows lower pneumonia and all-cause death rates with influenza plus pneumococcal vaccination versus influenza alone^12^. Additionally, a US Medicare cohort with beneficiaries ≥65 years suggested PCV13 reduced pneumonia hospitalization, independently of influenza vaccination^13^. Similarly, a retrospective cohort study with data from health insurance of almost 140,000 German adults (≥60) suggested that influenza vaccination can prevent severe complications from influenza, and pneumococcal vaccination can prevent hospital-treated pneumonia. However, they have not found this effectiveness for influenza plus pneumococcal vaccination^14^.

This study did not demonstrate that pneumococcal or influenza vaccines alone reduced pneumonia-related hospitalizations or all-cause death. However, influenza vaccination in older adults is well established to lower influenza-related hospitalizations^15^ and deaths^16^. Pneumococcal vaccines have also proven effective: PPSV23 reduced hospital stay, mortality, and costs in a Japanese cohort^17^, and in the United Kingdom, it lowered pneumonia risk by 15% (ages 65–74) and 40% (age ≥80)^18^.

The present study found that pneumococcal plus influenza vaccinations reduced pneumonia hospitalizations without affecting all-cause death. These may reflect the study population’s characteristics, including advanced age and multiple comorbidities, which could introduce residual confounding factors. Furthermore, the concerning low vaccination rate may have influenced these results. Healthcare professionals are incentivized to stay up to date with the vaccine data and can receive performance-related bonuses. Nevertheless, this low vaccination rate may be due, in part, to incomplete registration. Even considering this possibility, the vaccination rate is below expectations. Global pneumococcal and influenza vaccination targets for older adults are 70–86%^19,20^. In Belo Horizonte, influenza coverage was 64.9% in 2021 and 78.8% in 2022, higher than rates in the studied population^21^. Data on pneumococcal coverage in Brazil are scarce, but in 2015, coverage among older diabetic patients in São Paulo was 59.2% for influenza and only 26.1% for pneumococcal vaccines^22^.

The present study has limitations inherent to its retrospective cohort design and use of secondary database sources. These include the possibility of selection and information bias, missing data, and the restriction to variables available in the database, which may lead to residual confounding. In addition, in Cox regression analyses, adjustment covariates were assessed at baseline, assuming proportional hazards and not accounting for changes over time. Furthermore, a causal inference cannot be reliably established. Other residual confounders cannot be ruled out, despite adjustments for the main potential confounding variables like age, multimorbidity, and frailty. Despite these limitations, this study includes the data of a significant sample of frail older adults, generating real-world evidence of this population. To our knowledge, this is the first large observational study on this subject in Latin America.

## CONCLUSION

In this real-world study of frail older adults, we found that receiving the influenza plus pneumococcal vaccine led to a 35% reduction in pneumonia-related hospitalizations.

## Data Availability

The datasets generated and/or analyzed during the current study are available from the corresponding author upon reasonable request.
